# Stepped collaborative care for pain and posttraumatic stress disorder after major trauma: a randomized controlled feasibility trial

**DOI:** 10.1080/09638288.2023.2254235

**Published:** 2023-09-14

**Authors:** Melita J. Giummarra, Sandra Reeder, Scott Williams, Anna Devlin, Rose Knol, Jennie Ponsford, Carolyn A. Arnold, Alex Konstantatos, Belinda J. Gabbe, Hance Clarke, Joel Katz, Fiona Mitchell, Elizabeth Robinson, Douglas Zatzick

**Affiliations:** a Department of Epidemiology and Preventive Medicine, School of Public Health and Preventive Medicine, Monash University, Melbourne, Australia; b Caulfield Pain Management and Research Centre, Caulfield Hospital, Alfred Health, Caulfield, Australia; c Central Clinical School, Monash University, Melbourne, Australia; d Department of Social Work, Alfred Health, Melbourne, Australia; e School of Psychological Sciences, Monash University, Clayton, Australia; f Monash-Epworth Rehabilitation Research Centre, Epworth Hospital, Richmond, Australia; g Academic Board of Anaesthesia & Perioperative Medicine, School of Medicine Nursing & Health Sciences, Monash University, Clayton, Australia; h Department of Anaesthesia, The Alfred Hospital, Melbourne, Australia; i Health Data Research UK, Swansea University Medical School, Singleton Park, Swansea University, Swansea, UK; j Department of Anaesthesia and Pain Management, Toronto General Hospital, University Health Network, Toronto, Canada; k Department of Anaesthesiology and Pain Medicine, University of Toronto, Toronto, Canada; l Transitional Pain Service, Toronto General Hospital, University Health Network, Toronto, Canada; m Department of Psychology, York University, Toronto, Canada; n Patient and Carer Coinvestigators with Lived Experience of Trauma, Australia; o Department of Psychiatry and Behavioral Sciences, University of WA School of Medicine, Seattle, WA, USA; p Harborview Injury Prevention and Research Center, University of Washington School of Medicine, Seattle, WA, USA

**Keywords:** Trauma, injury, hospitalization, brief intervention, pain, pTSD, recovery

## Abstract

**Purpose:**

To examine feasibility and acceptability of providing stepped collaborative care case management targeting posttraumatic stress disorder (PTSD) and pain symptoms after major traumatic injury.

**Materials and methods:**

Participants were major trauma survivors in Victoria, Australia, at risk of persistent pain or PTSD with high baseline symptoms. Participants were block-randomized, stratified by compensation-status, to the usual care (*n* = 15) or intervention (*n* = 17) group (46% of eligible patients). The intervention was adapted from existing stepped collaborative care interventions with input from interdisciplinary experts and people with lived experience in trauma and disability. The proactive case management intervention targeted PTSD and pain management for 6-months using motivational interviewing, cognitive behavioral therapy strategies, and collaborative care. Qualitative interviews explored intervention acceptability.

**Results:**

Intervention participants received a median of 7 h case manager contact and reported that they valued the supportive and non-judgmental listening, and timely access to effective strategies, resources, and treatments post-injury from the case manager. Participants reported few disadvantages from participation, and positive impacts on symptoms and recovery outcomes consistent with the reduction in PTSD and pain symptoms measured at 1-, 3- and 6-months.

**Conclusions:**

Stepped collaborative care was low-cost, feasible, and acceptable to people at risk of PTSD or pain after major trauma.

**IMPLICATIONS FOR REHABILITATION**
- After hospitalization for injury, people can experience difficulty accessing timely support to manage posttraumatic stress, pain and other concerns.
- Stepped case management-based interventions that provide individualized support and collaborative care have reduced posttraumatic stress symptom severity for patients admitted to American trauma centers.
- We showed that this model of care could be adapted to target pain and mental health in the trauma system in Victoria, Australia.
- The intervention was low cost, acceptable and highly valued by most participants who perceived that it helped them use strategies to better manage post-traumatic symptoms, and to access clinicians and treatments relevant to their needs.

## Introduction

Survival rates and functional outcomes following traumatic injury have significantly improved in recent years [1]. However, recent research has shown that two thirds of people continue to report persistent or worsening mental health or pain problems post-injury [2], and most do not receive timely treatment for those problems [3], especially people living outside major cities [4,5]. Moreover, pain and mental health conditions, especially posttraumatic stress disorder (PTSD), often co-occur after injury [6,7]. Several theories have been proposed to explain the cooccurrence of pain and PTSD. The mutual maintenance model proposes that shared factors maintain both chronic pain and PTSD, for example, due to attentional biases toward threatening internal or external stimuli, as well as heightened anxiety or the tendency to have catastrophic beliefs about pain [8]. Similarly, shared vulnerability models propose that heightened sensitivity to anxiety-related symptoms, in addition to unhelpful thoughts about pain and somatic symptoms, increase the risk of both pain and PTSD [7,9]. While there is inconsistent evidence for the directional relationships between pain and PTSD symptoms after injury proposed in these models [10], there is evidence that both outcomes appear to influence each other over time [11] perhaps due to common mechanisms (e.g., central sensitization and anxiety sensitivity [12]). As such, it appears that treatments that target the physiological, psychological and social factors associated with both pain and PTSD are warranted [13].

Despite their common co-occurrence, few interventions that concurrently treat both pain and PTSD have been developed and robustly evaluated. Instead most interventions tend to primarily focus on PTSD symptom management [14,15], which have been found to lead to significant reductions in PTSD symptom severity but have little impact on pain severity or disability [16]. For instance, there is strong evidence to support the effectiveness of stepped care case management interventions for the prevention of PTSD after hospitalization for injury [14], such as the Trauma Survivor Outcome Support (TSOS) intervention [17,18]. In these interventions, patient risk factors, concerns and symptoms are assessed within days or weeks of injury and a case manager provides psychoeducation, psychologically-based therapeutic support and referrals for treatments adapted to the individual patient. This type of intervention enables “stepped” increases in treatment complexity in response to patient needs and preferences over time [18], with the most intensive period of support typically in the first 3–6-months post-injury [14]. Importantly, compared with traditional session-based psychological or multidisciplinary treatments (e.g., Cognitive Behavioral Therapy) stepped care case management interventions are relatively low cost and have been found to generate benefits for a larger proportion of the patient population at risk of PTSD following injury, including people with significant social, psychological and health-related complexities [14].

Unfortunately, there are few pain-focused interventions for people who have sustained serious injuries [19], and most studies have focused on less severe injury (e.g., early physiotherapist-led interventions [20,21]) even when they do address psychological risk factors [22]. One approach that has been implemented for the prevention of post-surgical pain, which may also be effective in the context of traumatic injury, is the Transitional Pain Service (TPS) model of care [23–27]. Transitional pain services identify patients with high psychosocial and clinical risk factors for persistent post-surgical pain, disability and opioid use. Treatment commences at the bedside post-surgery, and provide psychologically-informed multidisciplinary pain management supports for up to 12-months. Importantly, transitional pain services focus on managing psychiatric concerns alongside pain [28], and have been found to reduce pain-related disability and opioid use post-surgery. However, this model of care has not been evaluated as an early intervention for pain management after serious injury.

Given the lack of interventions targeting both pain and PTSD the present study adapted these two existing models of care that primarily target mental health [18] or pain [23] to develop a holistic stepped collaborative care intervention for people at risk of persistent pain and/or PTSD post-injury. In particular, we integrated elements of the TPS model of care into the TSOS stepped collaborative care intervention, incorporating pain-specific risk screening criteria and therapeutic elements for pain management that can be offered alongside mental health supports (e.g., relaxation strategies, resuming and increasing activity levels, pain physician assessments or advice for optimizing analgesia and behavioral strategies for pain management). The strategies integrated into the intervention were also consistent with recommendations in current guidelines for acute pain management, including review of analgesics, provision of targeted reassurance and advice on the resumption of usual activities, and facilitation of access to physical and psychological therapy to support recovery [29,30].

As this is a new way of providing early intervention targeting both pain and mental health after traumatic injury, particularly in the context of an Australian major trauma service, the present study was undertaken to examine the feasibility, acceptability and reach of the intervention compared with usual care. We also sought to identify what further adaptations may be required to enhance successful implementation. Finally, we aimed to provide a preliminary examination of whether the intervention led to reduced PTSD symptoms and pain-related disability compared with people who did not receive the intervention. However, as it was a feasibility study we did not set out to recruit a sample that would enable us to reliably detect significant effects on symptoms.

## Materials and methods

This was a feasibility mixed methods evaluation of a trial that randomly allocated participants to study groups, and used blinded assessors to collect baseline and outcome data. The evaluation used concurrent triangulation, whereby qualitative and quantitative data were collected in parallel, and the insights from each method were used to validate the study findings [31].

All study procedures were approved by the Alfred Health Human Research Ethics Committee (Project 274/19). The intervention was an enhanced TSOS intervention that integrated processes from the Toronto TPS model of care with input from a steering committee of interdisciplinary clinical experts, trauma system stakeholders and patient partners with lived experience of trauma and disability. The patient partners also provided input throughout the project at steering committee meetings and one-on-one meetings with the lead investigator. The study protocol was registered to the Australian Clinical Trial Registry (ID: 12619001341112).

### Setting and data linkages

The study was conducted at The Alfred, Melbourne, Australia. Demographic characteristics, symptoms and treatment details were collected from electronic medical records and using questionnaires that were completed in hard-copy, online or over the phone with a blinded assessor. Symptoms and treatment use were recorded in a REDCap database at baseline (pre-randomization), and at 1, 3, and 6 months post-randomization [32,33]. Case management and case conferencing clinical notes were also recorded in the REDCap database. Primary outcomes were the study reach, recruitment and retention rates, intervention acceptability, and symptoms of PTSD and pain severity and disability. Secondary outcomes were depression, anxiety, drug and alcohol use, neuropathic pain, pain catastrophising, health status, functional impairments, and health service and medication use.

Study data were linked with some of the demographic, injury and health characteristics and 6-month outcomes data from the Victorian State Trauma Registry (VSTR). The VSTR is a population-based registry that collects demographic, health, injury and treatment information for all major trauma patients admitted to trauma-receiving health services across the state of Victoria, Australia [34]. The VSTR data are collected from hospital records and coders, and through structured telephone interviews with patients or proxy respondents at 6-months post-injury. Study data were also linked with Medicare Benefits Schedule (MBS) and Pharmaceutical Benefits Scheme (PBS) records of pain, mental health and allied health treatments and medications in the 12-months prior to injury up to 6-months post-injury. The MBS and PBS dataset included all consultations, procedures, tests and medications subsidized by the Australian Government.

In Victoria, Australia, people who sustain road transport or workplace injuries and are admitted to hospital are automatically eligible for a compensation claim to support their ongoing healthcare through the Transport Accident Commission or Worksafe Victoria, respectively, regardless of who was at fault. Payments made for treatments by the compensation schemes were not available for data linkage when this study was undertaken.

### Participants

Patients were recruited from November 2019 to September 2020. Electronic medical records for all probable major trauma admissions were screened against the demographic, injury and risk factor eligibility criteria (Table 1). Patients who met the eligibility criteria were invited to participate by one of the baseline assessors, and were asked to provide informed consent before completing the baseline assessment of symptoms. Only people with elevated pain or PTSD symptoms as defined below were then eligible to enroll in the study. We aimed to recruit a minimum of 30 participants per group (*N* = 60), which exceeded the sample size typically required for feasibility studies (i.e., a total of 30–40 patients) [35,36]. Extenuating circumstances due to the onset of the COVID-19 pandemic in the first 4 months of the trial significantly impacted recruitment as face-to-face contact with admitted patients was not permitted after March 2020 [37]. Therefore, a pragmatic approach was taken to recruit as many patients as possible *via* telephone for the duration of the funding for the trial.

**Table 1.** Risk criteria for PTSD or pain.

Outcome	Risk criteria
PTSD	Three or more: Pre-existing PTSD diagnosis at admission Any other psychiatric diagnosis at admission Any substance use disorder diagnosis or positive blood alcohol content or illicit drug test on admission Current or prior tobacco use Intentional injury Intensive care unit admission with mechanical ventilation* One or more prior trauma hospital admissions A death occurred in the injury event Funding source: compensable injury* Non-male gender Non-white ethnicity Aged 35–65 years* Socioeconomic disadvantage (e.g., unemployed, at risk of job loss, living in a disadvantaged neighborhood [i.e., Index of Relative Socioeconomic Advantage and Disadvantage state decile <5])*
Pain	One or more: Neuropathic injury Prior psychiatric or substance use condition Prior chronic pain Prior treatment for opioid-addiction disorder Intense post-injury or post-surgical pain (e.g., persistent intense pain requiring breakthrough analgesia beyond expected level for injury, acute pain consultations for severe pain, or discharge is delayed due to complex pain) High post-injury opioid consumption (i.e., ≥90 mg/day oral morphine, long acting opioid prescription) High levels of pain-related distress

* These risk criteria were added or adapted based on Australian research of risk factors for poor mental health or pain outcomes after injury.

#### Demographic and injury eligibility criteria

Patients were eligible if they were aged >16 years at time of injury, survived to hospital discharge, spoke English, and sustained major trauma as per VSTR major trauma criteria (i.e., provisional ISS > 12; urgent surgery for intracranial, intrathoracic or intra-abdominal injury, or for fixation of pelvic or spinal fractures; or ICU admission for more than 24 h with mechanical ventilation); and could be enrolled within 28 days of injury or discharge from ICU.

Patients were ineligible if they did not have a Medicare number; had no capacity to give consent due to moderate-severe brain injury (i.e., Glasgow Coma Scale score of 3–12 and head Abbreviated Injury Scale score > 2, or Post-Traumatic Amnesia >24 h) or cognitive impairment (e.g., due to delirium, Alzheimer’s Disease or a score of ≥ 5 on the Short Portable Mental Status Questionnaire) [38]; were injured from intentional self-harm, during acute psychosis, while committing a crime or while incarcerated; had a history or violence or were under police guard in hospital; or if they had no usual place of residence or means for maintaining contact. Approximately 4-months into recruitment the eligibility criteria were updated to exclude patients aged ≥65 years injured from a low fall as it was apparent that they were likely to have different support needs to other eligible patients. Moreover, people injured in a low fall have relatively lower rates of both pain and mental health problems [39], and typically transition to lower problems following injury [40] compared with people injured in a motor vehicle collision.

#### Risk factor and symptom eligibility criteria

The risk criteria for the development of persistent symptoms are outlined in Table 1, which is based on previously published criteria for PTSD [41] and post-surgical pain risk [23] for the TSOS and TPS interventions, respectively. Patients who met those criteria were then eligible if their baseline scores also indicated clinically elevated posttraumatic stress symptoms (i.e., PTSD Checklist-Civilian score ≥ 35), pain severity or interference (i.e., Pain Enjoyment of life and General activity score ≥4), neuropathic pain (i.e., DN4 Neuropathic Pain Diagnostic Questionnaire total ≥3), pain cognitions (Pain Catastrophizing Scale >30), or depression symptoms (Patient Health Questionnaire total >14).

### Randomization

Participants were randomized in blocks of 2–4, stratified by compensation status, after completing the baseline assessment. Randomization allocations were generated by an independent statistician using Stata, and were placed in opaque envelopes that were opened after completion of the baseline assessment. A safety protocol was followed for participants who disclosed suicidal thoughts on the PHQ-9 in any interview, regardless of group, including directly contacting their care providers to ensure that they had ongoing support.

### Enhanced usual care control condition

All participants received enhanced usual care that included a summary letter after the baseline assessment, and patient-focused leaflets from relevant organizations (e.g., Phoenix Australia, Pain Australia, Chronic Pain Australia, the Better Health Channel, Road Trauma Support Services) to provide information to aid understanding of their mental health and/or pain symptoms, potential self-management strategies and supports. The lead investigator liaised with the participant’s care team in the hospital or community, including participants who only received enhanced usual care in the control group, for any participants who reported significant unmanaged pain, distress or suicidal ideation at baseline to ensure they had access to ongoing care. The same investigator would also follow up any participant, and their primary care provider, if they expressed suicidal ideation during any follow up interview.

### Risk-stratified stepped collaborative care intervention

Participants randomized to the intervention group received usual care and the enhanced TSOS intervention. The design of the enhanced TSOS intervention is summarised in Table 2, which documents the rationale behind the intervention, how it was delivered and by whom in line with the Template for Intervention Description and Replication (TIDieR) checklist [42].

**Table 2.** Intervention design, consistent with the TIDieR checklist.

TIDieR domain	Description of the TIDieR component to be documented
The rationale, theory, or goal of the elements essential to the intervention	Pain and PTSD often co-occur after traumatic injury, and there is a need for early interventions targeting both outcomes. To prevent the persistence of mental health symptoms following traumatic injury there is strong evidence of broad population reach and effectiveness of stepped collaborative care case management interventions [14]. However, similar models of care have not been developed to prevent persistent pain, except in the context of post-surgical pain [23–27]. These interventions are effective because they include: Screening to identify people at risk of persistent symptoms and targeted intervention delivery only towards those who are at risk Ongoing assessment to identify when interventions need to be enhanced or optimized (i.e., “stepped” up) Tailoring of strategies and supports to the individual’s needs and preferences while also considering their resources to access or pay for supports in the community Ensuring people have appropriate primary care and allied health supports in the community to provide ongoing care. The goal of the intervention is to prevent persistent pain and mental health symptoms, particularly posttraumatic stress disorder.
Name of intervention	The enhanced Trauma Survivor Outcome Support (TSOS) intervention with integrated elements from the Toronto Transitional Pain Service (TPS)
Intervention materials	The TSOS intervention manual formed the primary basis of the intervention, which is available from the TSOS principal investigator Professor Zatzick. This was enhanced with strategies outlined in the design of the TPS, which is available from the TPS investigators Professors Katz and Clarke. The main therapeutic components of the intervention were: Elements of cognitive behavioral therapy, behavioral activation, relaxation, grounding strategies and psychoeducation The use of evidence-based smartphone applications to help participants to use self-management strategies (e.g., PTSD Coach) A motivational interviewing counselling approach for people with low engagement or risk behaviors, particularly for those engaging in substance use. Advocacy and support to access rehabilitation or therapy in the community, or through the relevant compensation scheme if they had a traffic or work-related injury.
Who provided the intervention	An experienced social worker who received training and weekly supervision in all aspects of the treatment (i.e., the adapted intervention manual for the TSOS intervention [17,18]), and the additional pain management processes in line with the TPS model of care [23] and acute pain management guidelines [29]. The broader clinical team included a supervising psychiatrist, a neuropsychologist, health psychologist and a pain physician.
Mode and location of intervention delivery	Participants were predominantly supported remotely through telephone or video calls. Written or printed resources were sent to participants using email or mail. Face to face case management support was also provided when appropriate and allowed in the context of COVID-19 physical distancing requirements, such as when participants were already scheduled to attend the hospital for outpatient appointments. The intervention was provided individually to the injured person, but could also include outreach to key family members and collaborative care with their healthcare providers in the community. Case management notes were recorded electronically in a REDCap database, which were discussed during case management supervision (weekly) and case conferencing meetings (every 3 weeks).
Duration, intensity and dose of intervention	Case management interactions were initially provided weekly or fortnightly, typically for 30–60 min per contact, depending on the participant’s symptoms, needs and preferences. Over time contacts were reduced in frequency for participants who reported that they no longer experiencing significant problems to the case manager, or in routine follow-up interviews; however, participants could always contact the case manager if they had new issues requiring support. The case manager met with the health psychologist and lead investigator for 30 min every week, and a 1-h case conferencing session was held every 3 weeks.
Tailoring of the intervention	Intervention components were fully tailored to the needs and preferences of each participant. A “stepped” care approach was used where the persistence or escalation of symptoms prompted the delivery of more regular or intensive supports. Decisions about therapeutic strategies or escalation of care were discussed at the routine case conferencing meetings or at ad hoc meetings if needed.
Adverse events	Any adverse events were recorded in case management notes and reported to the ethics committee and other parties, where relevant, to ensure the ongoing safety and wellbeing of participants.

Participants worked with a case manager who was supported by a broader clinical team that comprised a supervising psychiatrist, neuropsychologist, health psychologist and pain physician. The broader clinical team did not have direct contact with participants, or with the participant’s healthcare providers in the community, unless the participant required a specialized assessment or treatment aligned with the clinician’s area of expertise. In those instances, the case manager would refer the participant to the clinician.

The case manager was an experienced social worker who received training and ongoing supervision in all aspects of the treatment that was adapted from the intervention manual for the TSOS intervention [17,18] to integrate therapeutic elements to optimize pain management in line with the TPS model of care [23] and acute pain management guidelines [29]. The clinicians who developed the TSOS and TPS interventions were investigators on the project team, which ensured that the key elements of both models of care were integrated. The case manager participated in clinical team meetings weekly with the lead researcher and health psychologist, and every 3 weeks with the broader collaborative care team. The weekly clinical team meetings involved discussing and reviewing participant needs, symptoms, concerns, and safety, as well as the effectiveness of strategies used to date. The collaborative case conferencing meetings every 3 weeks provided the case manager with further coaching from the broader clinical team, and focused on discussion of barriers to treatment and the identification of new strategies or referrals to relevant health professionals and organizations, and/or potential medication changes to manage symptoms. The recommendations identified in the case conferencing meetings were then communicated back to the participant and their general practitioner later that day.

The case manager’s role was multifaceted. They primarily maintained regular contact with participants *via* phone, as well as video calls and emails. Case management contacts focused on establishing rapport through empathic and nonjudgmental communication. They also monitored symptoms, concerns or stressors; escalated care if symptoms persisted or increased; provided education about sleep, stress, pain, and trauma symptoms and explored suitable strategies and treatments. They supported care coordination and provided advocacy to improve access to treatments, income support or equipment; and empowered participants to self-direct their care and engagement with treatment. Where necessary, the case manager administered brief assessments if symptoms appeared to escalate in order to provide targeted care and support (e.g., the five item Primary care PTSD screen for DSM-5 to assess PTSD symptoms or an 11-point numeric rating scale for pain intensity).

The case manager used psychologically-informed strategies when supporting participants to manage their pain and mental health symptoms, including related issues like sleep or social isolation which can impact on both pain and mental health. A *motivational interviewing counselling approach* was used with participants exhibiting risk behaviors (e.g., drug or alcohol use) or who had low readiness to engage in treatment, and to encourage participants to direct their own recovery behaviors. *Cognitive behavioral therapy* elements, including behavioral activation, relaxation and grounding, were used to explore and challenge unhelpful thoughts or beliefs about the injury, psychological symptoms and pain. Through *behavioral activation*, participants were encouraged to schedule and participate in valued and/or pleasurable activities while also building their engagement in everyday activities to reduce the severity of pain through mobilization. *Relaxation and grounding techniques* were used to help participants manage overwhelming pain or PTSD symptoms, sometimes using evidence-based smartphone applications for guided practice when appropriate (e.g., PTSD Coach).

Safety plans were established for participants who reported suicidal ideation or worthlessness using the template developed by Beyond Blue [43], which they were encouraged to share with their family and healthcare providers. In their safety plan, participants documented warning signs that suicidal thoughts are building (e.g., feeling hopeless or a burden); reasons they have to live (e.g., family); how to make their space safe (e.g., limiting access to alcohol); activities to do that keep them safe or distract them from suicidal thoughts (e.g., enjoyable hobbies); people to talk to from their social network (e.g., a specific friend or partner); and professional supports they can contact (e.g., doctors, crisis helplines). By 6-months post-injury, the case manager aimed to have empowered participants to self-direct their care (e.g., reviewing questions they could ask their doctor), or linked them with social services if ongoing case management support was needed.

Support was tailored towards the unique needs of each participant based on their risk profile, treatment readiness and preferences, life circumstances, and existing treatment or rehabilitation. All treatments aimed to correspond with national and international evidence-based guidelines for post-trauma or post-surgical care [e.g., 44,45]. Notes were recorded for every case management and case conferencing encounter in REDCap, summarising the participant’s primary concerns, the strategies recommended or used, and the application of any strategies previously discussed.

### Materials

Demographic characteristics included age at injury, sex, preferred language, ethnicity, country of birth, education level; work status and occupation, income level and neighborhood characteristics at the time of injury; and relationship status, family structure, and living arrangements. Residential location was classified as major cities versus regional and remote [46]. Education level was classified according to the Australian Standard Classification of Education [47].

Prior comorbidities were collected from the Electronic Medical Record and in the baseline interview. Disability level in the week before injury was assessed using a five-level rating scale ranging from no disability to severe disability from the VSTR follow up interviews [48]. The revised US National Comorbidity Survey trauma history screen [17,49] assessed lifetime trauma experience including: experiencing or witnessing life threatening injuries, abuse or neglect (e.g., transport injury, interpersonal violence, sexual assault), disasters or exposures (e.g., toxic chemicals), illnesses, and war-related trauma.

Injury characteristics included the Injury Severity Score (ISS), injured body regions, length of hospital stay and discharge destination. The ISS is calculated using the sum of the square of the three highest Abbreviated Injury Scale (AIS) severity scores from three different body regions [50]. Injuries were classified as multi-trauma; orthopedic and thoracic/abdominal injuries; or neurotrauma. Injury causes were classified as injuries from road trauma & motorized vehicles, falls (including falls from horses), and other (e.g., burns, assault). The fund covering the injury related treatments (e.g., Medicare, transport or workers compensation) and access to private health insurance were recorded.

#### PTSD symptoms

PTSD symptom severity was measured using the PTSD Checklist – Civilian Version (PCL-C) [51], and four additional items consistent with the DSM-5 diagnostic criteria from the PCL-5 scale [52], as per previous studies [17]. The PCL-C was used as we implemented the validated risk screening criteria from another similar trial [17]. The PCL-C comprises 17 items, each describing a symptom of PTSD in the DSM-IV. Each symptom was rated from 1, “not at all,” to 5, “extremely,” and scores were summed to produce a total score reflecting overall symptom severity. Clinically elevated PTSD symptoms are indicated by scores ≥35 [41], and clinically meaningful change in PTSD symptoms is defined as a change of >10 points on the PCL-C [53].

#### Pain symptoms

Primary pain outcomes were the severity and impact of pain, measured using 11-point Numeric Rating Scales for pain intensity right now and in the past week using the Pain severity, interference with Enjoyment of life and General activity (PEG) tool. The PEG was developed in a primary care setting as an ultra-brief measure of pain and pain-related impacts [54], and has been used to assess cancer pain [55], and to evaluate interventions following traumatic injury [18]. The PEG comprises three items from the Brief Pain Inventory (average pain severity; interference of pain with enjoyment of life; interference of pain with general activity in the past week) rated from 0 “no pain” to 10 “pain as bad as you can imagine.” Scores are averaged to generate an overall score, with scores of ≥4/10 indicating moderate-severe pain [56,57]. Participants were asked to confirm whether their pain was related to their injury. A clinically significant change is defined as a change of one or more points [58].

#### Other outcomes

Additional symptoms and outcomes assessed included: neuropathic pain [DN4 Neuropathic Pain Diagnostic Questionnaire; 59], pain catastrophizing [Pain Catastrophizing Scale; 60]; symptoms of generalized anxiety [7-item Generalized Anxiety Disorder scale; 61] and depression [9-item Patient Health Questionnaire; 62]; substance use [NIDA modified-ASSIST screening tool; 63]; alcohol use [3-item Alcohol Use Disorders Identification Test; 64]; and health status [five-level EQ-5D; 65] and functional impairments [12-item World Health Organisation (WHO) Disability Assessment Schedule 2.0; 66]. Self-reported health care and medication use related to the injury since the previous assessment were recorded. Healthcare use included hospitalizations, inpatient or outpatient rehabilitation, primary care visits, and appointments with a psychiatrist, psychologist, counsellor, social worker, nurse practitioner, pain specialist, occupational therapist, physiotherapist, spiritual advisor, or other practitioner.

### Primary outcomes: feasibility and acceptability assessment

The primary outcomes were the feasibility and acceptability of the intervention in accordance with the Reach, Effectiveness, and Implementation elements of the RE-AIM framework [67]. *Reach* was assessed as the proportion of the eligible population who enrolled in the study, and the demographic, injury and risk factor characteristics of those who enrolled. Intervention *acceptability* was assessed as: (1) participant satisfaction with “overall health care,” healthcare for “personal or emotional problems” and for “pain problems,” each rated on a 5-level Likert rating from very dissatisfied to very satisfied; and (2) participant reaction to participation (“Had I known in advance what participating would be like for me I still would have agreed to participate,” rated on a 5-level Likert scale from True to False).

In-depth examination of acceptability was undertaken through qualitative interviews. All intervention group participants were invited to participate in a semi-structured interview *via* telephone or zoom about their experience with the intervention at 3–6 months after enrolment in the study. A trained researcher conducted all interviews, which were recorded and professionally transcribed for analysis. One interview was transcribed in real-time by an observer for a participant who preferred not to be recorded. Field notes during and after interviews were used to aid data analysis. A semi-structured interview guide (Supplementary Materials), informed by the Theoretical Framework of Acceptability (TFA) for healthcare interventions [68], explored acceptability of the intervention and its components from the participant’s perspective using open-ended questions to probe key topics in detail, while maintaining flexibility for discovery of topics [69,70]. The TFA includes seven key domains: affective attitudes (feelings about intervention), burden (perceived effort of participation), coherence (understanding of intervention and how it works), ethicality (whether it fits with one’s values), opportunity costs (benefits, profits, or values given up to participate), perceived effectiveness (whether it appeared to achieve its purpose), and self-efficacy (confidence to adhere with the intervention). Thirteen interviews were sufficient to address the study aims and analytical framework [71], as no new topics arose during analysis.

As a feasibility trial, the study was not intended nor powered to statistically demonstrate *effectiveness* on symptoms. However, the evaluation investigated potential signals on PTSD symptoms on the PCL-C and pain and interference ratings on the PEG. Analyses examined (1) the proportion of participants in each group with clinically significant reductions in the PCL-C and PEG, and (2) the effect size for reductions in the PCL-C and PEG between groups over time. Differences in willingness to engage in treatment between groups over time were also examined as an indication of effectiveness, including willingness to “talk to a counsellor or other mental health professional,” “take medications to help with your emotional symptoms/problems” or “take medications to help with your pain symptoms/problems,” each rated from 0 “not at all” to 10 “totally willing.”

*Implementation* considerations focused on (1) treatment access as reported in follow-up interviews and *via* linked data; (2) case manager ratings of participant engagement; (3) treatments delivered or facilitated by the case manager; (4) the types of concerns supported by the case manager; (5) the case manager’s workload (hours providing case management); and (6) the cost of case manager contact time at the pay rate for an experienced allied healthcare worker ($AUD 54.17 p/h). The case manager rated participant engagement for each case management encounter as “not engaged” (e.g., unable to contact), “somewhat engaged” (e.g., sporadically returns calls or attends appointments), “moderately engaged” (e.g., returns calls and consistently able to contact) and “extremely engaged” (e.g., excessive contact with or dependence on the case manager).

### Data analysis

#### Qualitative data analysis

Qualitative interview transcripts were managed and coded in Nvivo 12 (QSR International, Doncaster, Australia). Case management notes were also analysed in Nvivo 12 to ascertain the implementation of the intervention and the use of its key components, reported in supplementary materials. The qualitative interview data were analyzed inductively using six non-linear stages of analysis of the framework approach [72]. Analysis stages included: (1) *familiarization* by reading transcripts to generate initial impressions; (2) line-by-line *coding* to identify and apply labels to specific behaviors, values, emotions, attitudes, and experiences in transcripts; and (3) identification of the thematic *framework* through iterative discussion between team members. Two coders independently read the same five transcripts to develop the preliminary framework through discussion with a third analyst [73]. The same two coders then analyzed all transcripts by (4) *indexing* to apply the thematic framework codes and categories to transcripts; (5) *charting* to summarize data in matrices to organize findings by codes and categories between- and within-cases; and (6) *mapping and interpretation* to compare and contrast codes, categories, and cases to identify patterns, relationships, differences, and themes to develop concepts, explanations, and strategies based upon the observed phenomena [73].

To promote trustworthiness of the findings and to minimize potential impacts of researcher biases, the principles of credibility, dependability, transferability, and confirmability were applied [74,75]. The internal validity and *credibility* of the data were supported through regular discussion during study design, data collection, analysis, and interpretation, and the use of peer review, debriefing, and framework co-development. *Dependability* was enhanced by documenting research processes with detailed notes on revisions of the topic guide, and coding frameworks to enable the rigor of the research to be audited. *Transferability* was enhanced by accounting for the context of the experiences (e.g., through detailed descriptions and use of quotes, using pseudonyms instead of participants real names). *Confirmability* was enhanced by examining the extent that the findings represented the dataset, rather than the researcher’s assumptions, biases, or conflicts of interest. Confirmability was further explored using negative case analysis to ensure that nuanced perceptions across the sample were captured, including non-normative responses of the sample [74].

The qualitative data collection and analysis was carried out by researchers with backgrounds in psychology and nursing. Two researchers had contact with participants during recruitment or case conferencing, and therefore had some knowledge of their experiences post-injury. Although pre-existing relationships could help build rapport with participants they could also introduce biases, which were addressed using peer debriefing and reflexive journaling [74,75].

#### Quantitative data analysis

The quantitative data were analyzed using Stata (Version 15, College Station, TX: Stata Corporation). Most analyses addressing the feasibility evaluation involved reporting of descriptive statistics, including number (percent), mean (*M*) and standard deviation (*SD*), or Median (Med) and interquartile range. Potential signal for the effectiveness of the intervention on willingness for treatment, and effects on PTSD symptoms and PEG scores were examined using mixed effects linear analyses, which included interaction effects between study group and time (baseline, 1-month, 3-months, 6-months), random intercepts for participant identity, and unstructured covariance. Due to the small sample size, no covariates were included and 100 bootstrapped samples were used to generate 95% confidence intervals.

## Results

### Sample characteristics and intervention reach

**Figure 1.**
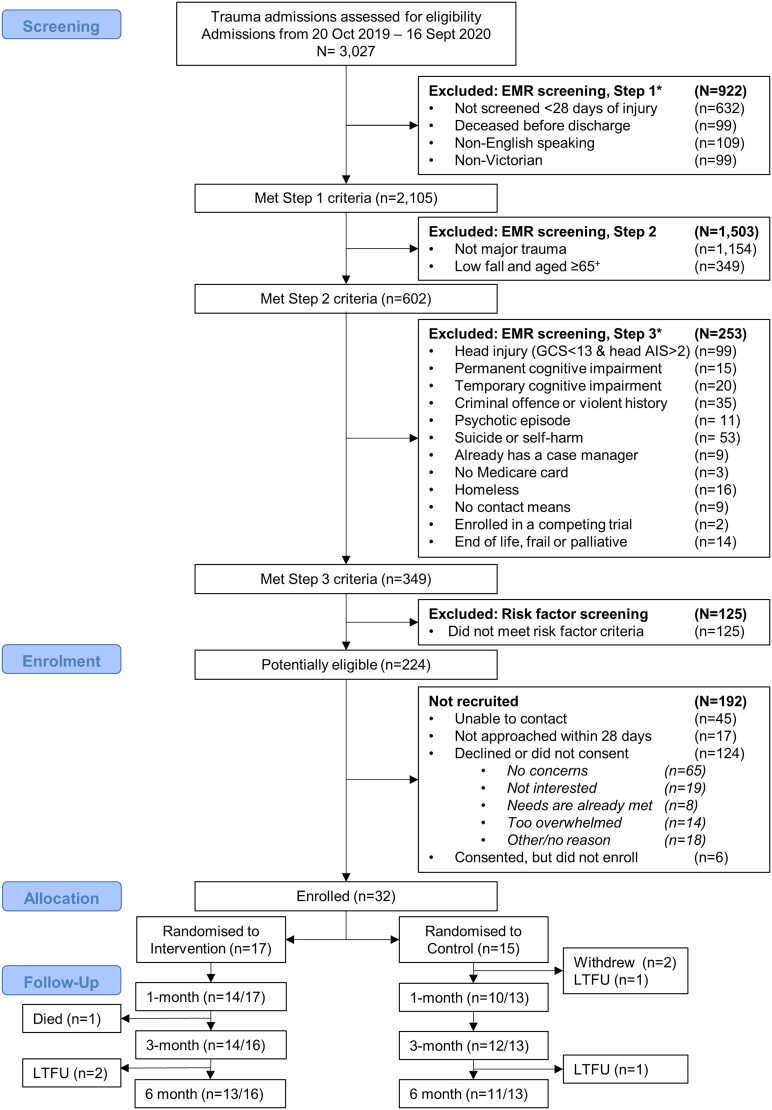
Participant inclusion flow chart. *Note. Some patients met multiple exclusion criteria, LTFU indicates participants completely lost to follow up from the respective time-point; + exclusion of patients aged 65 years and older injured in low falls was introduced in March 2020.

Two hundred and twenty four patients were potentially eligible, of whom 162 were invited to participate in the baseline assessment (Figure 1). Ninety two patients reported that they had no concerns, that their needs were already being met or that they were not interested in receiving support and were therefore not likely to be at risk of persistent problems [76]. Of the remaining 70 patients likely to need support, 32 (45.7%) consented and completed baseline asssessments, all of whom had elevated pain and/or PTSD symptoms at baseline and enrolled in the study. The most prevalent risk factors of participants who enrolled in the study were: middle age (*n* = 19, 63%), compensable injury (*n* = 17, 57%), smoking history (*n* = 15, 50%), non-male gender (*n* = 12, 40%), and low socioeconomic status (*n* = 12, 40%). Ten participants were intoxicated at the time of injury or used illicit substances pre-injury (predominantly cannabis [*n* = 5] and/or stimulants [e.g., methamphetamine, *n* = 4]), and seven (23%) had a prior psychiatric diagnosis. Seven participants (23%) were admitted to ICU.

Sample characteristics are summarised in Table 3. Participants were predominantly male (*n* = 18, 60%), had a median age of 48 years (Q1 = 33, Q3 = 57), and had low levels of education (i.e., an advanced diploma or lower than secondary school (*n* = 19)). Most participants were married or living with a partner (*N* = 22, 76%), were living in major cities (*n* = 21, 70%), and were working prior to injury (*n* = 23, 76%). Participants reported excellent pre-injury health (EQ-VAS, Med = 95, Q1 = 85, Q3 = 100); however, 18 (60%) participants had pre-existing comorbidities, and there were high levels of prior trauma (Med = 5 traumatic experiences each; Q1 = 3, Q3 = 6). The only characteristics that differed significantly between groups at baseline was a higher prevalence of participants with one or more comorbid conditions in the intervention (76%) versus control group (38%), and higher baseline depression symptoms in the control group (Med = 17, Q1 = 11, Q3 = 19) compared with the intervention group (Med = 9, Q1 = 3, Q3 = 12).

**Table 3.** Sample characteristics at baseline.

		Intervention group (*N* = 17)	Enhanced usual care (*N* = 13)
Demographic, health and prior trauma			
Age (years), Med (Q1, Q3)		54 (41, 61)	47 (33, 51)
Age group (years), *n* (%)	18–34	4 (24)	5 (38)
	35–54	6 (35)	5 (38)
	55+	7 (41)	3 (23)
Gender, *n* (%)	Male	10 (59)	8 (62)
	Female	7 (41)	5 (38)
Ethnicity, *n* (%)	Anglo Celtic/Caucasian	11 (73)	8 (73)
	Other (European, Asian, Maori)	4 (27)	3 (27)
Prior Health (EQ VAS), Med (Q1, Q3)		96 (87, 100)	90 (80, 95)
Prior trauma (number), Med (Q1, Q3)		4 (3, 7)	6 (3, 6)
Injury characteristics			
Blame someone for the injury at baseline, *n* (*%*)		9 (53)	10 (77)
Blame self		6 (40)	4 (44)
Blame another		1 (6.7)	3 (33)
Injury Severity Score, *M* (*SD*)		17 (10)	17 (5)
Cause of injury, *n* (%)	Road trauma & motorized vehicle impacts	7 (41)	7 (54)
	Falls	3 (18)	4 (31)
	Other	2 (15)	7 (41)
Fund, *n* (%)	Medicare	9 (53)	4 (31)
	Transport Accident Commission	5 (29)	5 (38)
	Workcover	3 (18)	4 (31)
Hospital LOS (days), Med (Q1, Q3)		10 (7, 16)	6 (5, 11)
Risk of developing PTSD or pain			
Pain risks, *n* (%)	One or more risk present	4 (24)	6 (46)
Total PTSD or pain risks, *n* (%)	<4/36 risks	9 (53)	5 (38)
	≥4/36 risks	8 (47)	8 (62)
Total PTSD risks, *n* (%)	<3/10 risks	7 (41)	6 (46)
	≥3/10 risks	10 (59)	7 (54)
Demographic risks, *n* (%)	Middle-aged	11 (65)	8 (62)
	Low SES or job at jeopardy	5 (29)	7 (54)
Baseline symptoms			
PTSD (PCL), Med (Q1, Q3)		35 (26, 50)	35 (25, 51)
Depression (PHQ-9), Med (Q1, Q3)		9 (3, 12)	17 (11, 19)
Pain (PEG), M (SD)		6.57 (2.28)	6.49 (2.01)
Suicidal (PHQ-9, Item 9), *n* (%)	Yes	5 (29)	6 (46)

*Abbreviations*. BAC: blood alcohol content; LOS: length of stay; *M*: mean; Med: median; *n*: number; PCL: PTSD Checklist; PEG: Pain severity; interference with Enjoyment of life and General activity; PHQ: Patient Health Questionnaire; PTSD: Posttraumatic Stress Disorder; Q: quartile; *SD*: standard deviation; SES: socioeconomic status; VAS: visual analogue scale.

Participants were predominantly injured in road trauma (*N* = 14) as motor vehicle or motorcycle occupants (*n* = 9), or pedestrians and pedal cyclists (*n* = 5). Other causes of injury were falls (*n* = 7), burns (*n* = 3), injuries involving horses (*n* = 3), and penetrating or blunt force trauma (*n* = 3). The median ISS was 17 (Q1 = 13, Q3 = 21), and participants spent a median of nine days in hospital (Q1 = 5, Q3 = 16). Injured body regions included chest and abdominal injuries (*n* = 11), followed by neurotrauma (*n* = 9), orthopaedic injuries (*n* = 7) and multitrauma (*n* = 3). Seventeen participants had one or more surgical procedures under anaesthetic (control group, *n* = 6, 50.0%; intervention group, *n* = 11, 64.7%).

No participants withdrew from the intervention group (*n* = 17), but two people were lost to follow up at 6-months post-injury, and one died from a health condition before the 3-month follow-up. Two participants withdrew from the control group in the first month (*n* = 15), and another two were lost to follow up at 1-month and 6-months. Follow-up assessment completion rates were high: 80.0% at 1-month, 89.7% at 3-months, and 82.8% at 6-months (Figure 1).

### Intervention group: content of case management and concerns post-injury

#### Participant contacts and engagement

The case manager spent a total of 166 h in contact with the 17 participants in the intervention group, at a median of 7 h and 4 min per participant (Q1 = 5.45 h, Q3 = 14.67 h). The average cost of the case management contact time, per participant, was $AUD383. For all participants there was a median of 19 (Q1 = 8, Q3 = 23) telephone or video interactions between the case manager and participants out of a median of 24 (Q1 = 21, Q3 = 26) contact attempts per participant (total of 379 contact attempts). Most contact was *via* telephone (*n* = 17; Med = 18; Q1 = 12, Q3 = 21), followed by video calls (*n* = 7; Med = 11; Q1 = 1, Q3 = 18). Email was used for five participants, predominantly to share information and resources after a telephone or video session.

Participants were rated as moderately engaged for most sessions (*n* = 288 sessions, 74.8%), somewhat engaged for 20.0% of sessions (*n* = 77 sessions), and not engaged for 5.2% of sessions (20 sessions). No participant was extremely engaged (i.e., excessive contact with or dependence on the case manager). Across sessions, individual participants were typically moderately engaged (*n* = 9) or somewhat engaged (*n* = 5) for the majority of contacts, and only three participants were not engaged or only somewhat engaged for most sessions (i.e., two participants were somewhat engaged for half of all sessions, and one was unable to be contacted or sporadically attended sessions).

#### Case management supports provided

The key elements of the intervention provided to participants are summarized in Table 4. All participants received care coordination, and behavioral activation was used with all participants, focused on various valued recreational activities, including: cooking; social connections; joining in celebrations, and religious, spiritual or meditation-based activities; re-engaging with education or training; resuming physical activity; gardening and home maintenance; and other interests and hobbies. Most other participants also received the other CBT elements, and were supported to identify and use strategies to manage symptoms. The motivational interviewing technique was used with five participants to address risk behaviors. Further description of how supports were provided is available in supplementary results.

**Table 4.** Intervention processes, *N* = 17.

		Number of encounters with process^a^	
	Number (%) of participants who received each process ≥ once	Mean (*SD*)	Med [Q1, Q3]
Care coordination			
Any care coordination	17 (100.0)	5.80 (4.29)	5 [3, 8]
Primary care provider	6 (35.3)	1.17 (0.41)	1 [1, 1]
Psychology or psychiatry	14 (82.4)	3.57 (2.50)	3 [1, 6]
Rehabilitation	10 (58.8)	1.80 (1.03)	2 [1, 2]
Pain specialist	5 (29.4)	1.40 (0.89	1 [1, 1]
Social services	2 (11.8)	4.00 (4.24)	4 [1, 7]
Addiction services	2 (11.8)	1.50 (0.71)	2 [1, 2]
Medication management			
Any medication-related support	16 (94.1)	3.13 (3.01)	2 [1, 3]
Current medications discussed	12 (70.6)	2.17 (1.70)	2 [1, 3]
A change in medications discussed	11 (64.7)	2.36 (2.16)	1 [1, 3]
CBT elements, any			
Any CBT elements	14 (82.4)	5.00 (4.33)	4 [1, 7]
Identifying solutions or strategies, including behavior activation	13 (76.5)	3.85 (2.88)	4 [1, 6]
Psychoeducation	7 (41.2)	2.14 (1.86)	1 [1, 3]
Relaxation or grounding	9 (52.9)	2.11 (1.90)	2 [1, 2]
MI elements	5 (29.4)	4.4 (2.70)	4 [3, 6]

^a^ Only including cases who had one or more session that included the respective element.

*Abbreviations*. CBT: Cognitive Behavioral Therapy; MI: motivational interviewing.

#### Harms or adverse effects

There were no intervention-related harms.

### Qualitative evaluation of intervention acceptability

Twelve participants and two partners of participants from the intervention group participated in 13 qualitative interviews (Med = 35.95 min per interview). Three key themes characterized the acceptability of the intervention, including (1) valued components of the intervention; (2) positive impacts of the intervention on symptoms, recovery and outcomes; and (3) suggestions for improvement and future development of the intervention.

#### Theme 1: valued intervention components

For several participants establishing rapport was essential to *developing a trusting relationship* (subtheme 1.1). The case manager was perceived to be nonjudgmental, and provided supportive listening through regular one-on-one interactions, all of which made participants feel comforted, heard and reassured:

It’s just natural that everyone gets a bit guarded … and the more you talk to someone you’re able to relate to them. (Adam, road trauma, metropolitan area)

Participants described how they experienced a trusting relationship because the case manager was “very kind, considerate, forthcoming” (Brian, work injury, living in a regional area), and someone they could “depend on, that really took a lot off your plate” (David’s partner, road trauma, living in a regional area). Participants further reported that the case manager was trustworthy and valuable because she “was quite knowledgeable in how to deal with these things” (Eric, road trauma, living in metropolitan area). The case manager’s support was considered particularly important over time for participants who felt they could not continue to discuss the injury with family or friends:

People who are close to you often assume you’re recovered – physically you look fine again, but people don’t see the hidden injuries and impacts. So, having someone there to validate that it’s ok that you’re still feeling that way or having some problems or concerns is really important. (Fiona, work injury, living in metropolitan area)

Trustworthiness was also reinforced by the fact that the case manager “never overstated her ability or confidence, but indicated she would consult with someone on the team who had the expertise or knowledge of service providers for specific concerns. That was really valuable.” (Fiona, work injury, living in metropolitan area).

Participants acknowledged that the intervention helped them to access *tailored and helpful services, supports, resources and strategies* (subtheme 1.2) with “very practical, pragmatic and specific advice” (Fiona, work injury, living in metropolitan area), such as learning and using new strategies to manage symptoms or concerns, and to identify and access new resources to support their recovery. Some participants noted that the case manager helped them to practice strategies they had learned from their psychologist or other healthcare providers, and that she ensured that she and their other healthcare providers were using consistent strategies to support their recovery and their safety:

It was really good, because she was, basically, reminding me of past strategies and forms that I’d used in the past, with medication and yoga, mindfulness, just calming aspects, which were really what I used in the past. (Eric, road trauma, living in metropolitan area)

[the case manager] has also been in contact with my mental health team where I live. She’s worked with them to try and help. Everyone is more or less on the same page. (Hannah, burns injury, living in regional area)

Participants recognized that the case manager advocated for them in a way that improved their access to care (e.g., with their rehabilitation providers or the compensation claim manager). Through consultation with the broader clinical team, participants also felt that the case manager could identify a wider range of appropriate services and supports. In particular, several participants stated that they initially had difficulty accessing this tailored support before they enrolled in the study:

[We] don’t know who to talk to or who to call… I tried it and you get bounced from place to place – oh no, this person, oh no, that person. So, it’s kind of frustrating in a sense to be on the outside. But someone that can do everything - that’s why I found it a lot easier once she took over. A lot of things got done. (Partner of David, road trauma, living in a regional area)

It was really good the help because otherwise no one would have really helped me out… It should be done for everyone. (David, road trauma, living in a regional area)

Other participants recognised that they received more appropriate support through the case manager than their own doctor could organise:

The psychologist the GP recommended wasn’t really appropriate. They just didn’t have much experience with trauma or injury. But the case manager had a whole team to go to and ask for recommendations, which felt like **my team** [spoken with emphasis], and I was able to get better advice and connection with services and a psychologist with experience in trauma. (Fiona, work injury, living in metropolitan area)

The case manager’s consistent, reliable, one-on-one support was delivered flexibly over time, which participants noted to be one of the most valued benefits of the intervention. Most participants reported that engaging with the case manager placed minimal burden on them, and that any effort required was worthwhile for the benefits received: “You’re talking to someone on the phone… it’s just a mental effort, and I got something out of it. So, I think… that makes the effort very little.” (Adam, road trauma, living in metropolitan area)

Several participants reported that they sometimes felt overwhelmed with the information they received from other healthcare providers and that the case manager helped them to understand when they had difficulty understanding their injury and what they needed to do to recover:

I couldn’t really understand some of the words that they [the doctors] were saying, and it was just more so … not dumbing it down, but just in terms that we could understand. Because, obviously, doctors have their terminology. So, it was just explaining it better, and just being there, to do everything… in a lot of cases, you’re dealing with people that haven’t had this injury before. What I found is very good, is the fact that there is someone that’s got a level of knowledge that can pass that information on to whether it be the patient or their carer. (David, road trauma, living in a regional area)

Some participants also described how the case manager supported them to learn how to coordinate their own care over time, which helped many participants to feel that they had were able to self-direct their care by the end of the study.

#### Theme 2: positive impacts on symptoms, recovery and outcomes

Most participants noted that the case management support helped them improve their ability to manage symptoms and concerns. One participant even lamented that “If I didn’t have this weekly support I don’t know what sort of state I would be in, because of the issues that I’ve been exposed to, and I’ve had to deal with, on top of a fairly serious accident” (Carolyn, horse-related injury, living in metropolitan area). When asked how much of an impact the case management and care coordination had on their recovery, many participants emphasized that it reduced their stress levels. For instance, one participant stated that “I didn’t have to stress so much because I’ve got a case manager, she does all the headache stuff*”* (David, road trauma, living in a regional area). Another reported that the case manager “sent out something to me… It was kind of like yoga techniques, and things like that… [to] calm your mind and things like that… it helps at the end of the day to relax me and prepare me for going to bed, and things like that, so I get a decent night’s sleep.” (Brian, work injury, living in a regional area)

For some people speaking with the case manager helped to shift their perspective about their injury, and improved their knowledge, awareness and recovery expectations:

I’m just thinking ‘What on earth am I doing wrong?’ So, I’m questioning myself. My anxiety goes through the roof. And then I start speculating, imagining, and whatever, but having the support in place with the case manager helps me put it more into perspective, and we talk through how to deal with how I’m feeling and how to deal with the situation. (Carolyn, horse-related injury, living in a regional area)

Others highlighted that the case manager instilled self-management strategies that participants could apply and use into the future. Several participants felt that they could not have managed without the case manager’s support. This was particularly the case for participants who were frustrated with a lack of support from the compensation scheme, and who felt that the case manager “provided me more support than what the compensation scheme has” (Gail, road trauma, living in metropolitan area). Several participants spoke of the need to manage grief related to the injury, which the case manager helped them to deal with.

#### Theme 3: areas for improvement and future development

Participants shared some *barriers to engagement* (subtheme 3.1). Some participants highlighted that it took time to establish trust and rapport when they were initially “a bit guarded” to disclose their concerns to the case manager (Adam, road trauma, living in metropolitan area). One participant avoided contact over time if they did not want to speak about the trauma, and preferred to manage their recovery on their own:

It’s not that I didn’t have the time, I just don’t want to talk about it again… I’ve been offered stuff, but I just do things by myself… I’m stubborn that way. (Ian, fall, living in metropolitan area)

The scheduling and availability of the case manager, who only worked 1–2 days per week, was occasionally reported as problematic if participants had support needs that emerged throughout the week. There were occasionally issues from technology and internet connections that sometimes impacted on the use of video calls with the case manager. Some participants said they would have preferred more face-to-face contact but could only use telephone or video contact due to pandemic-related restrictions. Moreover, the scheduled telephone interviews to assess symptoms over time caused additional distress for one participant who sustained a mild brain injury and had difficulty concentrating, but who could easily complete the assessments online. While this reflected a difficulty related to the participation in the evaluation, rather than the intervention, per se it has important implications for future trial design:

Having trouble doing the telephone interview actually had a negative impact on me. It raised more concerns about my capacity, and because I was struggling with the telephone interviews it impacted on my questions about whether I was doing ok or going to recover ok. (Fiona, work injury, living in metropolitan area)

Participants offered *suggestions for future case management support delivery* (subtheme 3.2). All participants said that they would recommend the support to others because it required minimal effort and had few disadvantages, particularly given the positive impacts it had on their wellbeing and recovery. Some hoped that case management would become routine for people recovering from serious injury. For instance, Lee stated that “it would be good if all patients of trauma cases should have access to this support… this will be very useful to all those patients similar to me” (work injury, living in metropolitan area). Others provided similar statements on the need for this support to be made routinely available:

[it should be] a standard service implemented in the hospital in the trauma unit ward… and then when you were at home, I think feeling like you could contact someone more than just that one time each week, knowing if it’s not your day but you’re struggling you could contact them when there’s a problem. (Fiona, work injury, living in metropolitan area)

I think it’s a really important program, and it needs to be rolled out permanently across Australia… it was good to have someone who has an understanding of the various aspects of medical fields… giving you mental, psychological and emotional support… I think it’s something that the government needs to provide financial support and ongoing resources to help fund this… if they didn’t roll this out nationally, they are doing more harm than good. (Joseph, fall, living in metropolitan area)

Some participants, especially people who were injured after March 2020 when the researchers were not permitted onto the ward due to COVID-19 restrictions, commented that it would have been better to start receiving case management support while they were still in hospital, and for support to be available flexibly throughout the week. However, other participants found it was challenging to engage with the case manager early post-injury as they were “pretty much in a drug haze” from medication side-effects, and when they had “so much going on, scheduled through the day” with their rehabilitation (Eric, road trauma, living in metropolitan area). Others who had more complex injury, health or social concerns wanted to extend the duration of case management beyond 6-months as they still experienced problems accessing supports and several felt that it was important that their partner or family members were involved. For one of those participants a new case manager in the community was arranged to provide ongoing support.

Several participants spoke about experiencing *additional issues that impacted on their life throughout the program* (subtheme 3.3), and that the case manager supported them with those concerns. The additional challenges included social isolation, having difficulties at work, or accessing treatment for new health concerns not related to the injury. For instance, Ian stated that because of both the pandemic and his injury “You never see people. It’s just been a hard couple of months, and I’ve been in the shit, which has been terrible.” (Ian, fall, living in metropolitan area)

### Quantitative outcomes between groups

#### Treatment willingness, use and satisfaction

**Figure 2.**
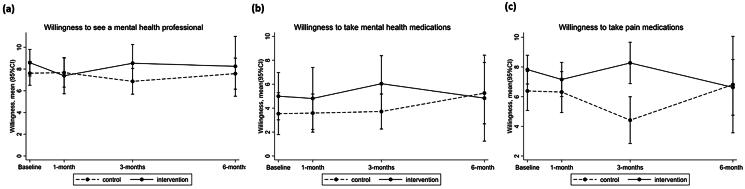
Willingness for treatment for mental health or pain symptoms, 95% confidence intervals estimated with 100 bootstrapped samples.

Participants in both the intervention and control group had high levels of willingness to see a counsellor or mental health professional, and moderate willingness to take medications for emotional symptoms over time (Figure 2). Over time there were no apparent differences between groups in treatment willingness (Supplementary Table 1), or the proportion of people who accessed medications (Supplementary Table 2) or treatments *via* clinicians for pain or mental health (Supplementary Table 3). It was feasible to record treatment use over time through both self-report and MBS and PBS data linkage; however, five participants declined to allow data linkage to their treatment records, and there were small inconsistencies between self-report and treatment records.

**Figure 3.**
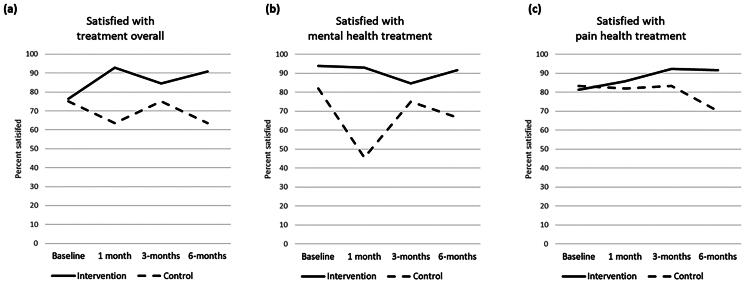
Percentage of participants satisfied or very satisfied with treatment over time.

Overall, most participants in both groups were satisfied or very satisfied with their overall healthcare and with the treatments received for mental health and pain (Figure 3). By the end of the study all intervention group participants said that they still would have participated had they known what was involved, but three participants in the control group reported that they would not have participated, and two other participants had withdrawn from the control group.

#### Symptoms

Descriptive statistics for the severity of all symptoms and levels of disability over time are reported in Supplementary Table 4.

##### PTSD symptoms

**Figure 4.**
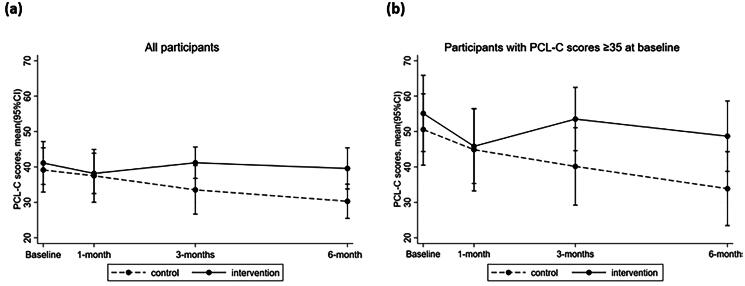
PCL-C scores for the control and intervention group over time in all participants, and in participants with high baseline PTSD symptoms.

Both the intervention and control groups had significant reductions in PTSD symptoms between baseline and 6 months post-injury (Beta = −8.85, 95%CI: −15.75, −1.94), with no differences between groups over time (Figure 4; Supplementary Tables 5 and 6). The proportion of participants who had a clinically significant change on the PCL (i.e., >10-point reduction compared with baseline ratings) did not differ between the intervention and control group at 1 month (29% vs 20% had >10 point reduction, *p* = 0.74), 3 months (43% vs 50% had >10 point reduction, *p* = 0.80) and 6 months (29% vs 40% had >10 point reduction, *p* = 0.68).

##### Pain symptoms

**Figure 5.**
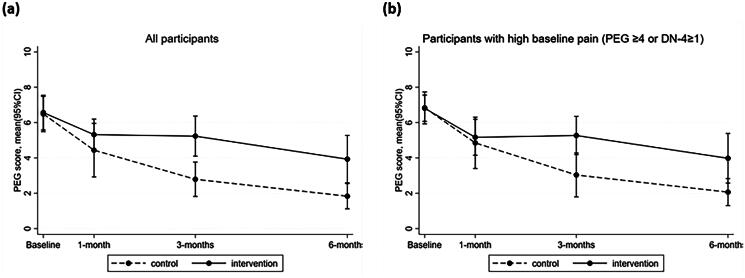
Pain (PEG) scores between the control and intervention group over time in all participants, and participants with higher baseline symptoms.

There were significant reductions in pain (i.e., PEG scores) between baseline and 1 month (Beta = −1.98, 95%CI: −3.79, −0.18), 3 months (Beta = −3.80, 95%CI: −5.43, −2.16) and 6 months post-injury (Beta = −4.77, 95%CI: −5.92, −3.61) for both the intervention (*n* = 17) and control groups (*n* = 13; Figure 5). Interaction effects showed that the intervention group had higher PEG scores than the control group at 3 months (mean difference = 2.44, 95%CI: 1.01, 3.88) and 6 months post-injury (mean difference = 2.10, 95%CI: 0.50, 3.69). Similar effects were observed for participants with higher baseline pain (30 of the 32 participants; Supplementary Tables 7 and 8, Figure 5).

For the 28 participants with clinically elevated pain at baseline there were no differences in the proportion of those participants with a clinically significant change on the PEG scores (i.e., >1 point reduction compared with baseline ratings) between the intervention and control group at 1 month (43% vs 64% had >1 point reduction, *p* = 0.30), 3 months (57% vs 83% had >1 point reduction, *p* = 0.80) and 6 months (77% vs 100% had >1 point reduction *p* = 0.09).

## Discussion

The present study demonstrated that we successfully adapted the stepped collaborative care intervention developed by Zatzick, et al. [18], and integrated principles from the TPS model of care [23], to provide case management support for both pain and mental health for major trauma patients admitted to a major trauma service in Victoria, Australia. The intervention successfully tailored supports to individual participant needs, with the key elements of the intervention being delivered to most participants. All participants in the intervention group received support for care coordination, most received support that used CBT-based principles including behavioral activation, and all received support or advice to use strategies tailored to their individual needs (e.g., relaxation training). Motivational interviewing was used with a small number of participants who had riskier drug or alcohol use, or who reported other risky behaviours. These strategies were successfully used in a way that participants felt that the case manager provided non-judgmental support.

Delivery of the intervention was feasible and highly acceptable for nearly all participants, and most had good levels of engagement. The qualitative data showed that participants appreciated receiving consistent and flexible access to support and advice from the case manager who was considered to be knowledge, reliable, and caring. Participants reported that they learned to use a range of strategies and accessed helpful resources or treatments through the case manager, and that the case manager provided important assistance to connect them with appropriate healthcare providers for their needs. The effort required by participants who were engaged with the intervention was reported to be worthwhile given the benefits that they experienced with respect to treatment access; advocacy with social, welfare, insurance, and compensation agencies; and improved capacity to manage symptoms or to use strategies to help them to function in everyday life. Participants unreservedly recommended that this type of case management should be made available to other patients like them.

The barriers in the intervention were predominantly related to the limited availability of the case manager throughout the week, which was necessary because the funding only enabled the case manager to work 1–2 days each week. Moreover, the COVID-19 pandemic had a marked impact on participants’ social isolation, and access to healthcare and other sources of support. Few participants had very low engagement, but those who did have lower engagement typically described a preference for managing on their own, or had difficulty engaging when there were several competing appointments. Some participants with low engagement also had very complex social circumstances, trauma histories or illicit substance use; however, it should be noted that these participants did still engage with the case manager in the first few months post-injury and received at least some intervention elements, and none withdrew. We would therefore urge other researchers and clinicians to continue to give all patients at risk of persistent pain or PTSD an opportunity to enroll in interventions like these, even if they are more likely to have low engagement given that they represent a relatively large proportion of the at-risk population.

While participants who received the intervention felt that it played an integral role in their recovery, both groups had similar reductions in PTSD symptoms over time, and the participants in the intervention group reported higher pain at 3–6 months. These findings may be spurious given that the sample size was not large enough to reliably detect effects of the intervention. Moreover, despite using blinded randomization, small samples can still have imbalances in group characteristics [77]. For the present study both groups were similar for some demographic and injury characteristics (e.g., both groups had similar injury severity and a similar proportion blamed another person for the injury); however, the intervention group included all of the participants who had burn injuries, intentional injuries and neurotrauma, which may explain the higher pain ratings in that group over time relative to the control group. Regardless of these potential differences, participants in both groups had high willingness for treatment and similar access to treatment, and satisfaction with treatment. The level of access to mental health treatments between groups may partially explain why both groups reported similar reductions in PTSD symptoms over time, whereas the apparent higher willingness for pain medications at 3-months in the intervention group was consistent with reports of higher pain severity in that group at 3- and 6-months. It should be noted, however, that the quantitative findings are only representative of this particular cohort given the small sample size. Nevertheless, the findings are consistent with effects reported in another study that evaluated the TSOS model of care in a much larger pragmatic trial across 25 level I trauma centers in the USA [17]. The latter study also found similar levels of treatment access in the intervention and control groups, and only small effects on PTSD symptoms at 6-months when considering all study sites together, but there were large treatment effects for patients at sites with better implementation of the case management model [17].

### Implications

The present feasibility study has generated several insights to inform future implementation of this enhanced TSOS collaborative care intervention for management of pain and mental health post-injury. In particular, we make several recommendations about the necessary resources, processes and supports to provide case management support in a way that flexibly meets patient needs. When delivering the intervention it is important that the case manager takes the time needed to build rapport and trust with participants, to identify concerns that emerge over time, and to find ways to manage those concerns through strategies and treatments that people were willing and able to use, and that they can afford. People who have experienced traumatic injury typically experience a range of social, psychological, rehabilitation and financial concerns, and most psychological support for patients in major trauma services in the current setting were provided by social workers. It was therefore most appropriate that the case manager was a social worker with experience working with people with mental health concerns, consistent with other stepped and collaborative care interventions [17,78,79]. Access to clinical supervision on a weekly basis, as well as consultation with the broader team *via* case conferencing every 3 weeks, were integral to enable the case manager to integrate clinical advice from a range of perspectives related to rehabilitation and psychiatric care (including pain medicine and addiction expertise) and psychology (including health psychology and neuropsychology expertise). In addition to orientation to the therapeutic approaches outlined in the TSOS manual, the case manager benefited from receiving ongoing support and supervision from the broader clinical team.

Overall, the intervention was low cost, with the case management time costing a median of $383 per patient for direct patient-facing care; however, additional costs would need to be accounted for the additional work undertaken by the case manager such as contact attempts, liaising with care providers, case conferencing, record keeping and maintaining professional development.

### Strengths

This study evaluated the feasibility of interventions that have already been found to have significant population reach and impact in other countries [14]. The project team included expert input from experienced clinicians, researchers and people with lived experience of trauma and disability, who altogether supported the adaptation of study processes and communications in order to improve the acceptability of the intervention to patients. We successfully enrolled patients into the study who had a range of social and clinical complexities and trauma-related concerns, 13 of whom would traditionally be excluded from trials of psychologically-informed interventions due to their substance use histories, prior psychiatric diagnoses or prior chronic pain. However, in the present study these people were purposely invited to participate as they are known to be at higher risk of persistent pain or PTSD post-injury, and they represent a significant proportion of the trauma population.

### Limitations

The present sample size was appropriate for a feasibility study, but it was too small to reliably detect significant quantitative differences between groups. We initially aimed to recruit up to 60 participants, which still would not have given sufficient power to detect effects on PTSD or pain symptoms. However, we reiterate here that demonstrating significant effects on symptoms should not be the purpose of a feasibility study [77]. The COVID-19 pandemic emerged in the first 3 months of the study, and impacted negatively on recruitment as we could not meet patients face-to-face on the hospital trauma ward, and patients were not always easy to contact *via* telephone within 28-days of injury. Within the sample recruited, there may have been some biases between groups that would not appear in a larger randomized controlled trial. Therefore, while the present findings may not generalize to the broader trauma population, particularly people with very severe injuries (e.g., serious traumatic brain injury) who were not eligible, we demonstrated that it is feasible to recruit patients with a broad range of injury causes, circumstances and severities with various pre-existing complexities. Moreover, the findings do not generalize to older people injured in a low fall who were excluded given that this patient cohort tends to recover better than people injured in other contexts, such as motor vehicle collisions [40].

As we only conducted qualitative interviews with participants in the intervention group to understand the acceptability of the intervention we were not able to explore the treatment access, care coordination and recovery experiences of participants in the control group who had similar levels of treatment access. Previous research exploring discharge processes at the same hospital where the present study was conducted highlighted that patients often experienced great difficulty in navigating their care and recovery post-discharge [80], and no routine case-management support had been implemented since then. Finally, during 2020, the trauma ward in this hospital implemented a more intensive model of care to provide increased access to allied health and acute rehabilitation while still in hospital [81]; however, this did not extend beyond the inpatient admission so would not explain the levels of treatment access in the control group.

## Conclusion

This stepped collaborative care intervention, provided for the first 6-months post-injury, was found to be feasible and acceptable to patients at risk of developing persistent pain or PTSD. Future effectiveness and efficacy studies should take on board the lessons learned in order to provide robust, flexible individualized support to reduce the incidence of PTSD and pain after injury.

## Supplementary Material

Supplemental Material
