# Supplementary material for: Stepped collaborative care for pain and posttraumatic stress disorder after major trauma: a randomized controlled feasibility trial
Source: Disabil Rehabil. 2023 Sep 14;46(16):3643–59. doi: 10.1080/09638288.2023.2254235 (PMC10937328; doi:10.1080/09638288.2023.2254235)
Supplement: Supplemental Material [file IDRE_A_2254235_SM0188.pdf]

Supplementary methods

Supplementary Methods

Health characteristics

Prior comorbidities were collected from the EMR and in the baseline interview, including: hypertension, heart, circulatory or blood diseases; neurological disorders or prior brain injury; pulmonary disease; AIDS/HIV; increased lipids; eye disorders or vision impairment; liver disease; gastro-intestinal disorders; diabetes; obesity; cancer; arthritis; renal disease; thyroid disorders; osteoporosis; chronic pain; chronic fatigue; or sleep disorders. Disability level in the week before injury was assessed using a five-level rating scale ranging from no disability to severe disability from the VSTR follow up interviews [1].

Secondary outcome measures

Secondary pain outcomes included neuropathic pain symptoms and pain catastrophizing. The Neuropathic Pain Diagnostic Questionnaire (DN4) comprises four items on sensory descriptors and signs related to a bedside sensory examination [2]. A self-report version omitting the sensory testing has been demonstrated to have excellent capacity to discriminate nociceptive and neuropathic pain [3], and comprises two questions about the quality of pain (burning, painful cold, and electric shocks) and its association with abnormal sensations (tingling, pins and needles, numbness, and itching). A positive response to each item is given a score of 1, and a total score of ≥3/7 is suggestive of chronic pain with neuropathic characteristics. The Pain Catastrophizing Scale (PCS) measures the tendency to have an exaggerated negative mindset in response to painful experiences [4]. It comprises 13 items, and respondents rated the degree to which they had certain thoughts and feelings when in pain (from 0 'not at all' to 4 'all the time'). All items are summed to create a total score, and a score of >30 is considered clinically elevated [5].

Symptoms of anxiety, depression and suicidal ideation were measured with the 7-item Generalized Anxiety Disorder scale [6], and the 9-item Patient Health Questionnaire (PHQ) [7]. The

presence of drug or alcohol use disorders were assessed with the NIDA modified-ASSIST screening tool [8], and the 3-item version of the Alcohol Use Disorders Identification Test (AUDIT-C) [9].

The World Health Organisation (WHO) Disability Assessment Schedule 2.0 (WHO-DAS 2.0) [10] was used to measure functional limitations, and comprises 12 items, rated on a 5-point scale, relating to six live domains: (1) understanding and communication, (2) getting around, (3) self-care (4) getting along with others (5) household and work activities, and (6) participation in society. Responses were summed to generate a total score using the "simple scoring" method [10]. If responses were missing the score for the other item in the same domain was assigned to the missing item in accordance with the scoring guidelines. Health status was measured using the five level EQ-5D questionnaire [11], which measures the level of problems across five dimensions: mobility, self-care, usual activities, pain or discomfort, and anxiety or depression. Each dimension was rated with respect to the level of problems experienced in reference the present day. Self-reported health service use and medication use were collected in the follow up interviews, including whether participants accessed counselling and which recovery concerns those sessions focused on.

Interview script and topic guide

During the interview, I will ask some questions about the case-management support you have received as part of this study during your recovery. That is, the support that our case-manager may have provided to you. Your feedback will help us make improvements to the supports we provide, as well as to see if this kind of support is suitable for patients after serious injury. Please do speak freely about your experiences, there are no right or wrong answers. Anything that you share will remain confidential and will not impact on your involvement with the rest of the study or the support you receive. Anything you share will not be disclosed to the case manager unless you express that that you would like me to do so, though general information may be shared in order to make improvements to the support she provides. You may withdraw from the study, or from this part of the study, during the interview, or choose not to answer any questions.

Before we continue:

Do you give permission for this interview to be recorded?

☐ Permission given ____ Interviewer initials

Do you acknowledge that you have received and read the Participant Information Sheet when you were enrolled in the study?

☐ Acknowledgement given ____ Interviewer initials

Before we begin the interview, are there any questions you would like to ask?

Do you acknowledge that you have had the opportunity to ask questions and are satisfied with the answers received?

☐ Acknowledgement given ____ Interviewer initials

Do you understand that information about you will be kept confidential and that the data will be presented in a manner that does not identify you in any publications or presentations?

☐ Acknowledgement given ____ Interviewer initials

Do you agree to participate in this study as it is described?

☐ Consent given ____ Interviewer initials

Interview questions and prompts	Topic
1. Can you please describe the case-management support provided to you (that is, the support that [insert case manager name] has provided to you)?	Coherence

What was the purpose of the case-management support?	
2. Has the case-management support been helpful for you in your recovery? (How/Why not?)	Perceived Effectiveness
3. How do you feel about the case-management support provided to you (the support provided to you by [insert case manager name])?	Affective Attitude
4. How relevant has the has the case-management support been to your needs and concerns around your recovery?	Ethicality
5. Can you describe any needs or concerns that weren't addressed or met by the case-management support?	
6. What are your thoughts on the timing of the case management support after your injury?	
7. What are your thoughts on the frequency and length of the sessions with [insert case manager name]?	
8. What was it like for you to participate in the project?	Burden
9. How much effort does it take for you to participate in the project?	
10. Can you describe any disadvantages you have experienced in participating in the project?	Opportunity Costs
11. Can you describe any barriers or challenges to engaging in the case-management support?	Self-efficacy
12. Do you have any suggestions on how the case-management support could be improved?	Final Questions
13. Would you recommend this this kind of case-management support to other patients recovering from serious injury?	
14. Is there anything else you would like to share about your experience with the project that I have not asked you about?	

That was the last question. Do you have anything else you would like to add? Do you have any questions?

Thank you for your time. I really appreciate your feedback, and it's really valuable to hear your experience in the study. It will be really helpful for us to evaluate the support and make improvements to make sure it is a positive experience for people. We will be in touch after we have finished evaluating the intervention, and will provide you with a summary of the results.

Case Management note analysis

The use of key intervention components (e.g., CBT, behavior activation, motivational interviewing processes, care coordination or medication management) between the case manager and participants were recorded for each encounter, which were analyzed using descriptive statistics. The case management notes were uploaded to Nvivo 12 (QSR International, Doncaster, Australia) to enable coding of participant's concerns discussed in each encounter with the case manager, and how the case manager supported them to manage those concerns.

Supplementary results

Post-injury concerns

The case management notes highlighted that the main concerns participants had were related to mental health concerns, exacerbation of pain, sleep problems, functional limitations, general health, functional limitations, general health, return to work experiences and financial stress, and social and relationship problems.

The mental health concerns participants experienced included heightened anxiety, anger, frustration, depression, sadness, helplessness, mood swings, intrusive thoughts, rumination, cognitive

functioning problems, blunted affect, dissociative states, and adjustment to the injury. Many participants described concerns about PTSD symptoms, including avoidance, anxiety about exposure to trauma reminders, nightmares and distressing dreams, re-experiencing, hypervigilance and hyperarousal. Over time, many participants received support to build their self-esteem and self-worth, and six had issues with blaming others for the injury or feeling guilty about their role in the injury event. Six participants expressed thoughts that they would be better off dead post-injury or had suicidal thoughts, all of whom were supported to develop a safety plan.

Most participants experienced an exacerbation of pain (n=11), which was often attributed to specific activities, feelings or processes such as feeling distressed, new surgical procedures, resuming activities or flare ups when reducing pain medications. Eight participants reported that their pain improved over time, or that they were coping well with their pain. Participants expressed a range of emotions about their pain, including anger, fear and worry that persistent pain indicated their injury was more severe or not healing.

All but one participant had *sleep* problems due to pain, anxiety, PTSD symptoms, or the impacts of the injury (e.g., a facial injury causing snoring or discomfort when wearing a neck collar). Sleep problems resulted in disruptive daytime fatigue for five participants. Sleep improved over time for many participants who reported sleep problems, but several required ongoing supports and strategies.

The case manager noted that seven participants expressed concerns about functional impairments, disability, delays in healing or accommodations needed due to the injury (e.g., temperature sensitivity following burns); however, seven (including four of the participants who previously spoke of being concerned about their impairments) acknowledged improvements in their functional capacity over time. Several participants had issues with weight or nutrition post-injury, or the management of complex health conditions associated with the injury event such as needing better access to expert epilepsy assessment and management.

Many participants were actively planning to return to work or were optimistic about doing so (n=11), four returned on limited duties during the study, and nine had concerns about their ability to continue to work or return to work throughout the intervention. Some participants felt that there was pressure to return before they were ready from either their employer or the compensation scheme. Five participants had significant stresses related to work that played a role in their recovery even though not all of those stresses were directly related to the injury. Seven participants experienced significant financial stress post-injury due to their inability to return to work or challenges accessing income replacement and welfare payments, which led to fear of homelessness for some who were unable to pay their rent.

Twelve participants received support to manage additional stressors from the case manager, including coping with court or legal proceedings, relationship problems, and coping with other stressors not related to the injury that impacted on overall distress (e.g., home insurance claims for storm damage). Ten participants had relationship problems post-injury, and many experienced loneliness and isolation. Eight participants indicated that they had strong support from at least one family member or friend. Many participants found the COVID-19 pandemic distressing, especially people who had chest and lung injuries or pre-existing diseases that heightened their risk of serious illness. The State-mandated lockdowns during the pandemic also directly reduced access to treatment for several participants, with several participants avoiding treatment or opting for telehealth to reduce risk of catching coronavirus.

Appraisals of the impact of injury

Throughout case management interactions, several participants talked about the injury event and treatment experiences during their acute admission and discharge (e.g., experiencing hallucinations from medications, fearing safety from other patients, and worrying about readiness to return home), and worried about what the injury will mean for the rest of their life. The case manager noted that several participants, even those with persistent distressing symptoms, were grateful for having

survived and that the injury was not worse. Some participants told the case manager that the injury had a “silver lining”, or that it prompted them to reevaluate their purpose in life. Several participants wanted to provide peer support to other people with injuries.

In some case management interactions participants shared their anger about potential risk of reinjury for themselves or others, and some were concerned about repeated threats of violence. Some participants worried about the impact of the injury on their appearance or long-term function, especially people whose injuries led to significant changes in their appearance (e.g., following burns injury), which required support and strategies to cope with and accept the injury. Some participants were concerned about being a burden on family members. Five participants experienced grief over losses that resulted from the injury (e.g., having to sell the horse they could no longer ride), and others wanted to reach out to others involved in the injury event (e.g., via the police) to learn more about what happened, or to make sure that others were not upset or feeling guilty.

Case management supports provided

Care coordination included liaising with participant’s primary care providers, and supporting participants to access mental health clinicians, pain clinicians and rehabilitation providers. Several participants required access to culturally or spiritually appropriate and financially accessible strategies and supports. For five participants the case manager provided advice, advocacy and support to help participants access services, supports or equipment through social insurers or compensation schemes, or to access income support through welfare schemes. Six participants had problems with receiving timely access to supports or treatments via their compensation scheme.

Support strategies described in the case management notes for mental health concerns primarily included the use of relaxation, grounding and cognitive reappraisal; mood or sleep diaries to identify distress triggers; and use of pacing and routines to manage energy and increase activity levels. Participants were encouraged to talk about their injury with people they trusted, to learn more about what happened in the trauma event from people who were present, and to build acceptance. The case

manager encouraged compliance with medications if they were prescribed, and coached participants through exposure therapy homework if they were using that technique with a psychologist. The case manager supported participants to build self-advocacy skills (e.g., attending appointments with prepared questions), and established ongoing case management in the community for participants with high support needs when they completed the study.

Strategies to manage pain were similar to those used for mental health symptoms, and primarily included relaxation and grounding strategies; diaries to monitor activities that increased their pain; pacing to reduce the likelihood of exacerbating pain through overuse and fatigue; cognitive reappraisal to shift participant perspectives on the meaning of pain and to reduce pain catastrophizing; distraction techniques (e.g., watching television, playing games, reading or listening to podcasts); or applying heat or massage.

For participants experiencing problems with sleep, the case manager typically provided psychoeducation about sleep hygiene and recommended a range of strategies (e.g., limiting screen use before bed, using meditation, mindfulness or relaxation techniques, listening to music, having timed naps or rests during the day).

Advice or coordination of care with addiction services was provided for some participants, together with the use of motivational interviewing techniques to address risk behaviors for six participants due to ongoing alcohol use, drug use, thoughts of self-harm and other compulsive behaviors.

Supplementary Table 1. Coefficients for willingness for mental health and pain treatments over time, with bootstrapped confidence intervals.

	Willingness to see a mental health professional	Willingness to take medications for emotional symptoms	Willingness to take pain medications
	Beta (95%CI)	Beta (95%CI)	Beta (95%CI)
Group			
Intervention vs Control	0.97 (-0.83, 2.77)	1.46 (-1.23, 4.16)	1.44 (-0.14, 3.02)
Month			
1 month vs baseline	0.06 (-1.75, 1.87)	0.05 (-2.35, 2.46)	-0.08 (-1.92, 1.77)
3 months vs baseline	-0.74 (-2.40, 0.92)	0.18 (-2.03, 2.39)	-1.97 (-4.00, 0.06)
6 months vs baseline	-0.03 (-1.78, 1.71)	1.72 (-1.21, 4.64)	0.43 (-2.87, 3.72)
Group X time interaction			
At 1 month	-1.26 (-4.09, 1.57)	-0.24 (-5.00, 4.52)	-0.59 (-2.71, 1.53)
At 3 months	0.69 (-1.88, 3.27)	0.86 (-3.12, 4.84)	2.42 (-0.33, 5.16)
At 6 months	-0.30 (-3.42, 2.83)	-1.88 (-6.49, 2.73)	-1.62 (-5.53, 2.28)

Supplementary Table 2. Clinician attendances based on administrative medicare records (MBS) and self-report

	Enhanced Usual Care		Intervention		
	MBS* (N=12)	Self-report (N=13**)	MBS* (N=13)	Self-report (N=13***)	Self-report (N=17)
General Practitioner					
12-24 months pre-injury	11	11****	11	11****	14****
12-months pre-injury	9		11		
Baseline	4	2	3	0	0
At 1 month	7	7	4	6	10
At 3 months	9	9	10	11	12
At 6 months	7	9	12	8	10
Mental Health clinician [a]					
12-24 months pre-injury	3	8****	2	9****	11****
12-months pre-injury	2		1		
Baseline	0	2	0	0	0
At 1 month	2	1	1	3	4
At 3 months	3	4	2	3	4
At 6 months	2	4	2	4	5
Allied health professional [b]					
12-24 months pre-injury	1	9****	1	9****	11****
12-months pre-injury	1		1		
Baseline	0	0	0	0	0
At 1 month	0	5	0	6	9
At 3 months	0	6	0	11	12
At 6 months	1	8	1	8	10

Abbreviations: MBS = Medical Benefits Schedule

Notes: * MBS data only available for 12 participants in the control group and 13 participants in the intervention group; ** the number of participants reporting clinician attendances did not differ when including the additional participant who had not consented to MBS linkage; *** descriptives provided for the 13 participants in the intervention group who consented to MBS data linkage; **** self-report of the number of people who had ever previously seen the respective type of clinician. [a] Mental health clinicians included psychologists, psychiatrists, social worker, counsellor, other mental health professional, substance abuse or chemical dependency treatment; [b] Allied health clinicians included physiotherapist, exercise physiologist, occupational therapist, nurse or nurse practitioner.

Supplementary Table 3. Medication use for each group based on administrative prescription records (PBS) and self-report

	Enhanced Usual Care (N=12)		Intervention (N=17)		
	PBS* (N=12)	Self-report (N=13**)	PBS* (N=13)	Self-report (N=13***)	Self-report (N=17)
Opioids					
12-24 months pre-injury	3	n/a	2	n/a	n/a
12-months pre-injury	1	n/a	2	n/a	n/a
Baseline	1	3	2	2	2
At 1 month	7	4	8	3	4
At 3 months	6	2	8	2	3
At 6 months	3	1	4	1	1
Neuropathic pain medications					
12-24 months pre-injury	1	n/a	0	n/a	n/a
12-months pre-injury	0	n/a	1	n/a	n/a
Baseline	0	0	1	1	2
At 1 month	0	3	1	2	3
At 3 months	3	0	3	3	3
At 6 months	2	1	4	2	2
Other analgesics					
12-24 months pre-injury	2	n/a	2	n/a	n/a
12-months pre-injury	1	n/a	0	n/a	n/a
Baseline	1	2	0	2	2
At 1 month	3	2	5	2	3
At 3 months	2	4	2	3	3
At 6 months	3	0	2	0	0
Antidepressants					
12-24 months pre-injury	2	n/a	1	n/a	n/a
12-months pre-injury	2	n/a	2	n/a	n/a
Baseline	1	1	1	2	3
At 1 month	1	0	3	1	1
At 3 months	0	0	3	1	1
At 6 months	3	1	4	3	3

Abbreviations: PBS = Pharmaceutical Benefits Scheme.

Notes: * PBS data only available for 12 participants in the control group and 13 participants in the intervention group; ** the number of participants reporting clinician attendances did not differ when including the additional participant who had not consented to MBS linkage; *** descriptives provided for the 13 participants in the intervention group who consented to PBS data linkage.

Supplementary Table 4. Symptoms and outcomes over time.

	Enhanced Usual Care *n* = 13	Intervention *n* = 17
	Med (Q1, Q3)	Med (Q1, Q3)
PTSD (PCL)		
Baseline	35.00 (25.00, 51.00)	35.00 (26.00, 50.00)
1 month	29.00 (20.00, 49.00)	36.50 (24.00, 47.00)
3 months	26.00 (20.00, 42.00)	39.00 (22.00, 57.00)
6 months	23.00 (18.00, 42.00)	38.00 (24.00, 51.00)
Pain (PEG)		
Baseline	6.67 (5.33, 7.33)	6.67 (5.33, 8.00)
1 month	4.00 (1.00, 8.67)	6.17 (4.33, 6.67)
3 months	2.83 (0.50, 5.17)	5.33 (3.00, 6.67)
6 months	2.33 (0.00, 2.67)	3.67 (1.67, 7.00)
Depression (PHQ)		
Baseline	17.00 (11.00, 19.00)	9.00 (3.00, 12.00)
1 month	7.00 (1.50, 13.00)	10.00 (1.00, 15.00)
3 months	5.50 (1.00, 9.50)	7.50 (3.00, 13.00)
6 months	4.00 (2.00, 9.00)	6.50 (2.00, 12.00)
Anxiety (GAD)		
Baseline	10.00 (5.00, 14.00)	10.00 (6.00, 16.00)
1 month	2.00 (0.00, 12.00)	7.00 (0.00, 14.00)
3 months	2.50 (0.00, 6.50)	8.50 (2.00, 13.00)
6 months	4.00 (0.00, 9.00)	3.50 (2.00, 9.00)
Neuropathic pain (DN-4)		
Baseline	3.00 (1.00, 5.00)	4.00 (3.00, 4.00)
1 month	1.50 (0.00, 4.50)	3.00 (1.00, 4.00)
3 months	0.00 (0.00, 2.00)	3.00 (1.00, 4.00)
6 months	0.00 (0.00, 4.00)	1.50 (0.00, 3.00)
Pain Catastrophizing (PCS)		
Baseline	26.00 (2.00, 31.00)	16.00 (4.00, 25.00)
1 month	4.00 (0.00, 17.00)	10.00 (3.00, 18.00)
3 months	3.00 (1.00, 12.00)	7.50 (6.00, 26.00)
6 months	1.00 (0.00, 7.00)	6.00 (1.00, 18.00)
Disability (WHODAS)		
Baseline	39.00 (31.00, 46.00)	38.00 (27.00, 43.00)
1 month	29.00 (25.00, 36.00)	30.00 (24.00, 35.00)
3 months	16.00 (14.00, 31.50)	26.00 (19.00, 31.00)
6 months	19.00 (14.00, 23.00)	25.00 (19.00, 30.00)

Supplementary Table 5. Coefficients for PTSD symptoms over time.

	All participants	Participants with high baseline PCL
	Beta (95%CI)	Beta (95%CI)
Group		
Intervention vs Control	1.96 (-6.53, 10.46)	4.54 (-9.70, 18.78)
Month		
1 month vs baseline	-1.66 (-10.36, 7.05)	-5.69 (-22.76, 11.38)
3 months vs baseline	-5.62 (-14.13, 2.89)	-10.42 (-23.99, 3.15)
6 months vs baseline	**-8.85 (-15.75, -1.94)**	**-16.70** (**-30.67**, **-2.73)**
Group X time interaction		
At 1 month	-1.28 (-13.65, 11.09)	-3.57 (-26.00, 18.85)
At 3 months	5.70 (-5.68, 17.08)	8.84 (-11.62, 29.29)
At 6 months	7.31 (-3.71, 18.34)	10.27 (-10.05, 30.59)

Supplementary Table 6. Post-hoc comparisons of PCL scores over time.

	All participants	Participants with high baseline PCL
	Mean diff. (95%CI)	Mean diff. (95%CI)
Control group		
1 month vs baseline	-1.66 (-10.36, 7.05)	-5.69 (-22.76, 11.38)
3 months vs baseline	-5.62 (-14.13, 2.89)	-10.42 (-23.99, 3.15)
6 months vs baseline	**-8.85 (-15.75, -1.94)**	**-16.70** (**-30.67, -2.73**)
Intervention group		
1 month vs baseline	-2.93 (-12.16, 6.29)	-9.26 (-23.62, 5.09)
3 months vs baseline	0.08 (-7.86, 8.03)	-1.58 (-16.94, 13.78)
6 months vs baseline	-10.57, 7.50)	-6.42 (-21.87, 9.02)
Intervention vs control group		
At baseline	1.96 (-6.54, 10.46)	4.54 (-9.70, 18.78)
At 1 month	0.69 (-8.66, 10.04)	0.97 (-15.23, 17.16)
At 3 months	7.66 (-0.43, 15.76)	13.38 (-0.17, 26.93)
At 6 months	9.28 (1.53, 17.02)	14.81 (0.32, 29.31)

Supplementary Table 7. Coefficients for pain symptoms (PEG scores) over time.

	All participants	Participants with high baseline pain
	Beta (95%CI)	Beta (95%CI)
Group		
Intervention vs Control	0.08 (-1.14, 1.30)	-0.02 (-1.21, 1.17)
Month		
1 month vs baseline	**-2.05 (-3.87, -0.23)**	**-1.98 (-3.79, -0.18)**
3 months vs baseline	**-3.70 (-5.07, -2.33)**	**-3.80 (-5.43, -2.16)**
6 months vs baseline	**-4.65 (-5.87, -3.43)**	**-4.77 (-5.92, -3.61)**
Group X time interaction		
At 1 month	0.80 (-1.55, 3.14)	0.34 (-1.77, 2.45)
At 3 months	**2.36 (0.46, 4.26)**	**2.26 (0.18, 4.33)**
At 6 months	**2.02 (0.06, 3.97)**	**1.94 (0.04, 3.84)**

Supplementary Table 8. Post-hoc comparisons of PEG scores over time.

	All participants	Participants with high baseline pain
	Mean diff (95%CI)	Mean diff (95%CI)
Control group		
1 month vs baseline	**-2.05 (-3.87, -0.23)**	**-1.98 (-3.79, -0.18)**
3 months vs baseline	**-3.70 (-5.07, -2.33)**	**-3.80 (-5.43, -2.16)**
6 months vs baseline	**-4.65 (-5.87, -3.43)**	**-4.77 (-5.92, -3.61)**
Intervention group		
1 month vs baseline	-1.25 (-2.62, 0.12)	**-1.64 (-2.84, -0.44)**
3 months vs baseline	**-1.33 (-2.81, 0.15)**	**-1.54 (-2.74, -0.34)**
6 months vs baseline	**-2.64 (-4.22, -1.06)**	**-2.83 (-4.39, -1.27)**
Intervention vs control group		
At baseline	0.08 (-1.14, 1.30)	-0.02 (-1.21, 1.17)
At 1 month	0.88 (-0.97, 2.73)	0.32 (-1.37, 2.01)
At 3 months	**2.44 (1.01, 3.88)**	**2.24 (0.52, 3.95)**
At 6 months	**2.10 (0.50, 3.69)**	**1.92 (0.41, 3.42)**

References

[1] Williamson OD, Gabbe BJ, Sutherland AM, Hart MJ. Does recall of preinjury disability change over time? Injury Prevention 2013;19:238-43.

[2] Bouhassira D, Attal N, Alchaar H, Boureau F, Brochet B, Bruxelle J, Cunin G, Fermanian J, Ginies P, Grun-Overdyking A and others. Comparison of pain syndromes associated with nervous or somatic lesions and development of a new neuropathic pain diagnostic questionnaire (DN4). Pain 2005;114:29-36.

[3] Bouhassira D, Lanteri-Minet M, Attal N, Laurent B, Touboul C. Prevalence of chronic pain with neuropathic characteristics in the general population. Pain 2008;136:380-7.

[4] Sullivan MJL, Bishop SR, Pivik J. The pain catastrophizing scale: Development and validation. Psychological Assessment 1995;7:524-32.

[5] Sullivan MJL. PCS: The Pain Catastrophizing Scale user manual. Montreal, QC: McGill University; 2009.

[6] Spitzer RL, Kroenke K, Williams JB, Lowe B. A brief measure for assessing generalized anxiety disorder: the GAD-7. Arch Intern Med 2006;166:1092-7.

[7] Kroenke K, Spitzer RL, Williams JB. The PHQ-9: validity of a brief depression severity measure. Journal of general internal medicine 2001;16:606-13.

[8] World Health Organization ASSIST Working Group. The Alcohol, Smoking and Substance Involvement Screening Test (ASSIST): development, reliability and feasibility. Addiction 2002;97:1183-94.

[9] Bradley KA, DeBenedetti AF, Volk RJ, Williams EC, Frank D, Kivlahan DR. AUDIT-C as a brief screen for alcohol misuse in primary care. Alcohol Clin Exp Res 2007;31:1208-17.

[10] Üstün TB, Kostanjsek N, Chatterji S, Rehm J. Measuring Health and Disability: Manual for WHO Disability Assessment Schedule WHODAS 2.0. Geneva, Switzerland: World Health Organisation; 2010.

[11] Herdman M, Gudex C, Lloyd A, Janssen M, Kind P, Parkin D, Bonsel G, Badia X. Development and preliminary testing of the new five-level version of EQ-5D (EQ-5D-5L). Qual Life Res 2011;20:1727-36.
